# Supplementary material for: Monitoring Moroccan Honeys: Physicochemical Properties and Contamination Pattern
Source: Foods. 2023 Feb 24;12(5):969. doi: 10.3390/foods12050969 (PMC10000722; doi:10.3390/foods12050969)
Supplement: Supplementary file 1 [file foods-12-00969-s001.zip › foods-2199633-supplementary.pdf]

Supplementary Material

Table S1. Optimized MRM transitions and parameters of analytical validation for *n*=139 organic contaminants under analysis, including pesticides (*n*=108), PCBs (*n*=18) and PAHs (*n*=13). CE, Collision Energy; R^2, coefficient of determination; LOD, Limit of Detection; LOQ, Limit of Quantification.

No.	Compound	Transition 1 (*m/z*)	CE 1 (V)	Transition 2 (*m/z*)	CE 2 (V)	R^2	LOD (ng/g)	LOQ (ng/g)
				Pesticides				
**1**	(±)-Indoxacarb	218 → 203	10	218 → 134	20	0.995	0.14	0.51
**2**	Bendiocarb	166 → 151	10	166 → 109	20	1.000	0.02	0.04
**3**	Carbaryl	144 → 115	20	115 → 89	20	0.996	0.08	0.21
**4**	Carbofuran	164 → 149	15	164 → 103	20	0.996	0.05	0.11
**5**	Carbophenothion	157 → 121	20	157 → 77	20	0.998	0.05	0.15
**6**	Diethofencarb	267 → 225	10	267 → 168	20	0.994	0.04	0.12
**7**	Ethiofencarb	168 → 107	10	168 → 77	25	0.997	0.08	0.23
**8**	Furathiocarb	194 → 105	20	194 → 165	15	0.980	0.10	0.29
**9**	Phenoxycarb	116 → 88	15	186 → 109	15	0.990	0.15	0.56
**10**	Pirimicarb	238 → 166	10	206 → 166	15	0.998	0.04	0.14
**11**	Mecarbam	296 → 196	10	296 → 168	10	0.992	1.14	4.21
**12**	Azoxystrobin	344 → 329	20	344 → 183	20	0.989	0.06	0.2
**13**	Boscalid	342 → 140	10	342 → 112	25	0.987	0.13	0.42
**14**	Bupirimate	208 → 165	15	108 → 140	15	0.994	0.93	3.61
**15**	Captafol	151 → 79	20	151 → 122	10	1.000	0.03	0.09
**16**	Captan	107 → 79	10	107 → 77	20	0.986	1.32	4.37
**17**	Cyproconazole isomer II	222 → 125	20	224 → 127	20	0.998	0.06	0.17
**18**	Diclobutrazol	270 → 159	10	270 → 137	25	0.955	0.12	0.38
**19**	Fenarimol	251 → 139	20	251 → 111	25	0.985	0.32	1.05
**20**	Fenhexamid	177 → 78	20	177 → 113	20	0.922	2.54	8.38
**21**	Fluodioxonil	248 → 127	20	248 → 154	25	0.972	0.42	1.31
**22**	Flusilazole	233 → 165	20	233 → 152	20	0.980	2.55	8.36
**23**	Imazalil	215 → 173	15	215 → 145	25	0.989	0.26	0.86
**24**	Kresoxim methyl	206 → 131	10	206 → 116	10	0.990	1.38	4.23
**25**	Metalaxyl-M	160 → 130	20	160 → 144	20	0.995	2.32	8.22
**26**	Mepronil	269 → 119	10	210 → 181	20	0.992	0.07	0.23
**27**	Penconazole	248 → 157	20	248 → 192	20	0.992	0.41	1.22
**28**	Prochloraz	180 → 138	15	180 → 69	20	0.982	0.82	2.92

No.	Compound	Transition 1 (*m*/*z*)	CE 1 (V)	Transition 2 (*m*/*z*)	CE 2 (V)	R^2	LOD (ng/g)	LOQ (ng/g)
**29**	Procymidone	283 → 96	10	285 → 96	15	0.996	0.13	0.48
**30**	Pyrimethanil	198 → 118	30	199 → 198	25	0.986	0.25	0.82
**31**	Quintozen	237 → 143	20	237 → 119	20	1.000	0.12	0.36
**32**	Tebuconazole	250 → 125	15	125 → 89	25	0.999	0.15	0.47
**33**	Tolchlophos methyl	265 → 250	20	265 → 93	24	0.972	0.35	1.17
**34**	Triadimefon	208 → 181	10	208 → 127	15	0.994	0.11	0.34
**35**	Trifloxystrobin	190 → 130	15	190 → 102	25	0.992	0.31	0.99
**36**	Vinclozolin	212 → 177	15	212 → 145	20	0.998	2.45	8.24
**37**	Amandryn	227 → 170	10	227 → 185	10	0.994	2.49	8.18
**38**	Atrazine	200 → 122	15	215 → 200	10	0.996	0.12	0.4
**39**	Diflufenican	266 → 183	25	246 → 218	25	0.999	5.09	14.5
**40**	Linuron	160 → 133	15	160 → 125	15	0.984	5.51	19.3
**41**	Methabenzthiazuron	164 → 136	15	127 → 109	20	0.992	4.85	15.8
**42**	Oxyfluorfen	300 → 223	20	252 → 170	25	0.978	0.32	1.29
**43**	Propazine	214 → 172	15	214 → 94	20	0.952	0.54	1.62
**44**	Propyzamide	173 → 145	15	173 → 109	25	0.999	0.11	0.33
**45**	Simazine	201 → 173	7	201 → 186	8	0.998	0.05	0.17
**46**	Terbuthilazine	214 → 104	15	214 → 132	10	1.000	0.12	0.38
**47**	Trifluralin	264 → 160	15	264 → 206	10	0.961	5.11	17.08
**48**	Buprofezin	175 → 132	15	175 → 117	20	0.989	0.18	0.65
**49**	Cyromazine	151 → 109	15	165 → 123	20	0.999	0.06	0.18
**50**	Pyriproxyfen	136 → 78	20	136 → 96	20	0.990	0.16	0.54
**51**	2,4′-DDD	235 → 165	20	237 → 165	20	0.996	0.09	0.31
**52**	2,4′-DDE	246 → 176	20	318 → 248	20	0.962	0.52	1.62
**53**	2,4′-DDT	235 → 165	20	237 → 165	20	0.996	0.21	0.65
**54**	4,4′-DDD	235 → 165	20	237 → 165	20	0.998	0.09	0.29
**55**	4,4′-DDE	246 → 176	30	318 → 248	30	0.996	0.16	0.55
**56**	4,4′-DDT	235 → 165	20	237 → 165	20	0.998	2.33	8.23
**57**	Alachlor	188 → 160	15	161 → 146	15	0.985	0.26	0.95
**58**	Aldrin	263 → 193	20	293 → 258	20	0.986	0.82	2.85
**59**	*cis*-Chlordane	373 → 266	20	373 → 264	20	0.992	0.19	0.62
**60**	Dicofol	250 → 139	20	250 → 215	10	0.998	0.12	0.36
**61**	Dieldrin	263 → 193	20	263 → 228	20	0.996	0.12	0.53
**62**	Endosulfan sulfate	272 → 237	15	274 → 239	15	0.998	0.09	0.31

No.	Compound	Transition 1 (m/z)	CE 1 (V)	Transition 2 (m/z)	CE 2 (V)	R^2	LOD (ng/g)	LOQ (ng/g)
**63**	Endosulfan α	241 → 206	25	241 → 170	25	0.998	0.15	0.46
**64**	Endosulfan β	195 → 160	10	195 → 125	20	1.000	0.07	0.23
**65**	Endrin	263 → 193	20	281 → 245	15	0.972	0.09	0.29
**66**	Methoxychlor	227 → 169	20	227 → 141	25	0.978	0.12	0.38
**67**	*trans*-Chlordane	373 → 266	20	373 → 264	20	0.980	0.14	0.42
**68**	α-HCH	181 → 145	10	219 → 183	10	0.985	0.04	0.14
**69**	β-HCH	181 → 145	15	219 → 183	10	0.986	0.28	0.94
**70**	γ-HCH	181 → 145	15	219 → 183	10	0.998	0.12	0.42
**71**	Acephate	136 → 94	10	136 → 119	8	0.987	0.35	1.13
**72**	Andhion	231 → 175	15	231 → 129	20	0.969	0.36	1.15
**73**	Azinphos ethyl	160 → 132	5	160 → 77	10	0.999	0.29	0.91
**74**	Chlorpyriphos	197 → 169	15	197 → 169	15	0.996	1.68	6.04
**75**	Chlorpyriphos methyl	286 → 93	25	286 → 271	20	0.984	0.76	2.64
**76**	*cis*-Chlorfenvinphos	267 → 159	20	269 → 161	20	0.999	0.08	0.27
**77**	Coumaphos	226 → 163	20	226 → 135	25	0.984	0.29	0.92
**78**	Diazinon	137 → 84	15	179 → 137	20	0.992	0.09	0.28
**79**	Dimethoate	125 → 79	20	125 → 79	8	0.999	0.11	0.35
**80**	Fenamiphos	303 → 154	15	303 → 195	10	0.986	0.09	0.29
**81**	Fenchlorphos	285 → 270	20	285 → 240	20	0.998	0.07	0.21
**82**	Fenitrothion	125 → 79	15	277 → 125	18	0.990	0.07	0.23
**83**	Fenthion	278 → 109	20	278 → 125	22	0.998	0.19	0.58
**84**	Fenthion Sulfone	310 → 105	20	310 → 109	30	0.970	0.69	2.55
**85**	Fenthion Sulfoxide	278 → 109	15	278 → 169	25	0.995	0.04	0.13
**86**	Malathion	173 → 99	15	173 → 117	15	0.992	0.09	0.28
**87**	Methidathion	145 → 85	10	145 → 58	20	1.000	0.07	0.20
**88**	Omethoate	156 → 110	10	156 → 79	30	0.994	0.12	0.35
**89**	Parathion methyl	263 → 109	15	263 → 246	6	0.999	0.06	0.16
**90**	Phenthoate	274 → 125	15	274 → 121	15	1.000	0.11	0.30
**91**	Phosalone	182 → 111	20	182 → 75	30	0.997	0.04	0.10
**92**	Phosmet	160 → 77	25	160 → 133	15	1.000	0.04	0.10
**93**	Phoxim	109 → 81	15	109 → 91	15	0.998	0.10	0.29
**94**	Quinalphos	146 → 118	15	146 → 91	30	0.999	0.07	0.18
**95**	*trans*-Chlorfenvinphos	267 → 159	20	269 → 161	20	1.000	0.11	0.29
**96**	Triphenyl phosphate	325 → 169	20	325 → 77	25	0.997	0.13	0.30

No.	Compound	Transition 1 (m/z)	CE 1 (V)	Transition 2 (m/z)	CE 2 (V)	R^2	LOD (ng/g)	LOQ (ng/g)
**97**	Carbophenothion	157 → 121	20	157 → 77	20	1.000	0.11	0.37
**98**	Pirimiphos-methyl	290 → 125	15	290 → 151	15	0.998	0.14	0.46
**99**	*cis*-Fluvalinate	250 → 55	15	252 → 55	20	0.990	0.39	1.18
**100**	*cis*-Permethrin	183 → 153	15	183 → 168	15	0.994	0.52	1.76
**101**	Cypermethrin isomer I	181 → 152	20	163 → 91	15	0.992	0.08	0.26
**102**	Cypermethrin isomer II	181 → 152	20	163 → 91	15	1.000	0.1	0.3
**103**	Cypermethrin isomer III	181 → 152	20	163 → 91	15	0.999	0.05	0.14
**104**	Deltamethrin	181 → 152	20	253 → 93	15	0.999	0.1	0.33
**105**	*trans*-Fluvalinate	250 → 55	15	252 → 55	20	0.994	0.1	0.32
**106**	*trans*-Permethrin	183 → 153	20	183 → 168	20	0.986	0.09	0.29
**107**	Λ-Cyhalothrin	181 → 152	25	197 → 141	10	0.988	0.11	0.37
**108**	Piperonyl butoxide	176 → 131	15	176 → 103	20	1.000	0.06	0.18
				PCBs				
**1**	PCB28	256 → 186	15	258 → 186	15	0.999	0.12	0.38
**2**	PCB52	290 → 220	15	292 → 222	15	0.999	0.09	0.30
**3**	PCB77	290 → 220	20	292 → 222	20	1.000	0.08	0.22
**4**	PCB81	290 → 220	20	292 → 222	20	1.000	0.08	0.24
**5**	PCB101	324 → 254	20	326 → 256	20	0.996	0.06	0.22
**6**	PCB105	324 → 254	20	326 → 256	20	0.989	0.12	0.35
**7**	PCB114	324 → 254	20	326 → 256	20	0.996	0.14	0.49
**8**	PCB118	324 → 254	20	326 → 256	20	0.999	0.09	0.32
**9**	PCB123	324 → 254	20	326 → 256	20	0.998	0.08	0.24
**10**	PCB126	324 → 254	20	326 → 256	20	0.982	0.34	1.15
**11**	PCB138	360 → 290	25	362 → 292	25	0.982	0.13	0.46
**12**	PCB153	360 → 290	25	362 → 292	25	1.000	0.24	0.92
**13**	PCB156	360 → 290	30	362 → 292	30	1.000	0.06	0.19
**14**	PCB157	360 → 290	30	362 → 292	30	0.978	0.74	2.75
**15**	PCB167	360 → 290	30	362 → 292	30	0.996	0.33	1.13
**16**	PCB169	360 → 290	30	362 → 292	30	0.996	0.14	0.34
**17**	PCB180	394 → 324	20	396 → 326	20	0.992	0.15	0.52
**18**	PCB189	394 → 324	25	396 → 326	25	0.998	0.04	0.14
				PAHs				
**1**	Acenaphthylene	152 → 126	30	152 → 102	30	0.990	0.11	0.24
**2**	Anthracene	178 → 152	25	176 → 150	25	0.990	0.13	0.38

**No.**	**Compound**	**Transition 1 (*m*/*z*)**	**CE 1 (V)**	**Transition 2 (*m*/*z*)**	**CE 2 (V)**	R^2	**LOD (ng/g)**	**LOQ (ng/g)**
**3**	Benzo[a]anthracene	228 → 226	30	228 → 202	20	0.994	0.12	0.41
**4**	Benzo[a]pyrene	252 → 250	35	252 → 226	20	0.996	0.09	0.28
**5**	Benzo[b]fluoranthene	252 → 250	35	126 → 113	10	0.990	0.28	0.86
**6**	Benzo[g,h,i]perylene	276 → 274	45	276 → 272	50	1.000	0.1	0.31
**7**	Benzo[k]fluoranthene	252 → 250	35	126 → 113	10	0.994	0.17	0.48
**8**	Chrysene	228 → 226	30	228 → 202	20	0.990	0.1	0.34
**9**	Dibenzo[a,h]anthracene	278 → 276	30	278 → 252	20	1.000	0.11	0.32
**10**	Fluorene	166 → 165	15	165 → 164	20	0.996	0.12	0.42
**11**	Indeno[1,2,3-cd]pyrene	276 → 274	30	137 → 136	15	0.997	0.09	0.38
**12**	Phenanthrene	178 → 152	25	176 → 150	25	0.999	2.42	8.02
**13**	Pyrene	202 → 200	20	202 → 152	30	0.996	2.38	8.21

Table S2. Main figures of merit of analytical validation and monitored ions for investigated plasticizers, including *n*=10 PAEs and *n*=8 NPPs. R^2, coefficient of determination; LOD, Limit of Detection; LOQ, Limit of Quantification.

No.	Compound	Abbreviation	Monitored ions (m/z)	R^2	LOD (mg/Kg)	LOQ (mg/Kg)
			PAEs			
**1**	Dimethyl Phthalate	DMP	163, 92,164	0.995	0.007	0.023
**2**	Diethyl Phthalate	DEP	149, 177, 176	0.992	0.005	0.017
**3**	Dipropyl Phthalate	DPrP	149, 150, 209	0.993	0.007	0.020
**4**	Dibutyl Phthalate	DBP	149, 150, 223	0.994	0.007	0.023
**5**	Diisobutyl Phthalate	DiBP	149, 150, 223	0.992	0.007	0.023
**6**	Butyl Benzyl Phthalate	BBP	149, 91, 206	0.988	0.037	0.121
**7**	Diphenyl Phthalate	DPhP	225, 226, 104	0.994	0.015	0.051
**8**	Dicyclohexyl Phthalate	DcHexP	149, 167, 150	0.998	0.027	0.087
**9**	Diheptyl Phthalate	DHepP	149, 99, 265	0.995	0.177	0.553
**10**	Di(2-ethylhexyl) Phthalate	DEHP	149, 167, 279	0.998	0.007	0.020
			NPPs			
**1**	Dimethyl Adipate	DMA	114, 101, 111	0.996	0.010	0.030
**2**	Diethyl Adipate	DEA	111, 157, 128	0.994	0.013	0.037
**3**	Benzyl Benzoate	BB	105, 91, 212	0.989	0.012	0.033
**4**	Dibutyl Adipate	DBA	129, 185, 111	0.985	0.023	0.068
**5**	Diisobutyl Adipate	DiBA	129, 185, 111	0.995	0.008	0.027
**6**	Di(2-ethylhexyl) adipate	DEHA	129, 112, 147	0.986	0.013	0.037
**7**	Di(2-ethylhexyl) terephthalate	DEHT	149, 112, 261	0.988	0.070	0.233
**8**	Di(2-ethylhexyl) sebacate	DEHS	185, 149, 112	0.993	0.018	0.053

Underlined ions were considered for quantitative analysis

Table S3. Optimized MRM transitions and parameters of analytical validation for *n*=9 BPs under analysis. R^2, coefficient of determination; LOD, Limit of Detection; LOQ, Limit of Quantification

No.	Compound	Abbreviation	Transition 1 Quantifier ion (m/z)	CE 1 (V)	Transition 2 Qualifier ion (m/z)	CE 2 (V)	R^2	LOD (ng/g)	LOQ (ng/g)
**1**	4,4′-Sulfonyldiphenol	BPS	249.27 → 107.9	25	249.27 → 92.0	30	0.9993	0.30	1.0
**2**	4,4′-Methylenediphenol	BPF	199.23 → 93.1	15	199.23 → 105.1	25	0.9911	0.45	1.5
**3**	1,1-Bis(4-hydroxyphenyl) ethane	BPE	213.26 → 198.0	20	213.26 → 176.9	30	0.9931	0.30	1.0
**4**	4,4'-(propan-2,2-diyl) diphenol	BPA	227.29 → 212.1	10	227.29 → 133.0	20	0.9907	0.45	1.5
**5**	4-[2-(4-hydroxyphenyl) butan-2-yl] phenol	BPB	241.31 → 212.0	20	241.31 → 211.0	15	0.9944	0.30	1.0
**6**	2,2-Bis(4-hydroxyphenyl) hexafluoropropane	BPAF	335.30 → 265.0	30	335.30 → 177,0	20	0.9995	0.30	1.0
**7**	1,1-Bis(4-hydroxyphenyl)-1-phenyl-ethane	BPAP	289.36 → 274.1	35	289.36 → 211,0	25	0.9954	0.45	1.5
**8**	1,1-Bis(4-hydroxyphenyl)-cyclohexane	BPZ	267.30 → 145.0	15	267.30 → 222.9	30	0.9984	0.45	1.5
**9**	1,4-Bis(2-(4-hydroxyphenyl)-2-propyl)benzene	BPP	345.46 → 330.1	25	345.46 → 314,9	10	0.9992	0.45	1.5

Table S4. Main figures of merit of analytical validation for *n*=15 inorganic elements under analysis. R^2, coefficient of determination; LOD, Limit of Detection; LOQ, Limit of Quantification.

No.	Inorganic element	R^2	LOD (μg/kg)	LOQ (μg/kg)
**1**	Na	0.9978	1.533	5.059
**2**	Mg	0.9990	0.433	1.429
**3**	K	0.9980	0.501	1.653
**4**	Ca	0.9981	1.322	4.363
**5**	Mn	0.9998	0.003	0.010
**6**	Fe	0.9996	0.015	0.050
**7**	Co	0.9997	0.001	0.003
**8**	Ni	0.9997	0.007	0.023
**9**	Cu	0.9994	0.005	0.017
**10**	Zn	0.9993	0.038	0.125
**11**	Cr	0.9995	0.007	0.023
**12**	Al	0.9993	0.067	0.221
**13**	As	0.9998	0.001	0.003
**14**	Cd	0.9999	0.001	0.003
**15**	Pb	0.9999	0.001	0.003

Table S5. Daily intake limits and Oral Reference Dose (mg/Kg_{bw}/day) for each element. TDI: Tolerable Daily Intake; TWI: Tolerable Daily Intake; BMDL01: Benchmark dose at 1% level of extra risk (lower confidence level); BMDL05: Benchmark dose at 5% level of extra risk (lower confidence level).

Contaminant	Adequate intake (mg/Kg_{bw}/day)	Guideline value and reference	RfD (mg/Kg_{bw}/day)	Reference
**DEHP**	0.05	TDI [1]	0.02	[8]
**DEP**	5	TDI [1]	0.8	
**DBP**	0.01	TDI [1]	0.1	
**DEHA**	0.30	TDI [2]	0.6	
**BPA***	4	TDI [3]	0.05	
**Al**	1	TWI [4]	0.1	
**Ni**	22	TDI [5]	0.02	
**Pb**	0.5	BMDL01 [6]	0.0035	
**As***	3	BMDL05 [7]	0.0003	

*Expressed as μg/Kgbw/day
